# Supplementary material for: DNA methylation protects cancer cells against senescence
Source: Nat Commun. 2025 Jul 1;16:5901. doi: 10.1038/s41467-025-61157-7 (PMC12216915; doi:10.1038/s41467-025-61157-7)
Supplement: Supplementary file 1 — Supplementary Information [file 41467_2025_61157_MOESM1_ESM.pdf]

Supplementary Information

DNA methylation protects cancer cells against senescence

Xiaoying Chen[1,*], Kosuke Yamaguchi[1,2,*,#], Brianna Rodgers[1], Delphine Goehrig[3], David Vindrieux[3], Xavier Lahaye[4], Matthieu Nolot[1], Laure Ferry[1], Sophie Lanciano[5], Nadine Martin[3], Pierre Dubus[6,7], Fumihito Miura[8], Takashi Ito[9], Gael Cristofari[5], Nicolas Manel[4], Masato Kanemaki[2], David Bernard[3], Pierre-Antoine Defossez[1,#]

1: Université Paris Cité, CNRS, Epigenetics and Cell Fate, Paris, France.
* contributed equally
# Authors for correspondence: yamako0801@icloud.com, pierre-antoine.defossez@cnrs.fr

2: Department of Chromosome Science, National Institute of Genetics, Research Organization of Information and Systems (ROIS), Yata 1111, Mishima, Shizuoka, 411-8540, Japan.

3: Equipe Labellisee La Ligue Contre Le Cancer, Centre de Recherche en Cancerologie de Lyon, Inserm U1052, CNRS UMR 5286, Centre Leon Berard, Université de Lyon, Lyon, France.

4: Institut Curie, PSL Research University, INSERM U932, Paris, France.

5: University Cote d'Azur, INSERM, CNRS, Institute for Research on Cancer and Aging of Nice (IRCAN), Nice, France.

6: Department of Tumor Biology, Centre Hospitalier Universitaire de Bordeaux, 33000, Bordeaux, France.

7: BRIC U1312, INSERM, Bordeaux Institute of Oncology, Université de Bordeaux, 33076, Bordeaux, France.

8: Life Science Data Research Center, Graduate School of Frontier Sciences, the University of Tokyo, Kashiwanoha 5-1-5, Kashiwa, Chiba 277-8561, Japan.

9: Department of Biochemistry, Kyushu University Graduate School of Medical Sciences, Fukuoka, Fukuoka, 812-8582, Japan.

Supplementary Figures

Supplementary Fig. 1: Lack of apoptosis in cells depleted of UHRF1 or DNMT1; rescue experiments support the role of DNA methylation maintenance in preventing senescence.

Supplementary Fig. 2: Senescence-associated transcriptional programs, cytokine secretion, and chromatin modifications after UHRF1 and/or DNMT1 depletion.

Supplementary Fig. 3: DNA demethylation-induced senescence is independent of p53 and p16/Rb: further controls and quantifications; investigation of the role of L1 repeats and of aneuploidy.

Supplementary Fig. 4: Further investigation of the MYC-TFAP4-p21 cascade.

Supplementary Fig. 5: Additional experiments on cGAS and STING in senescence induced by loss of DNA methylation.

Supplementary Fig. 6: Validation and senescence assessment of HCT116 AID2 system *in vitro* and *in vivo*.

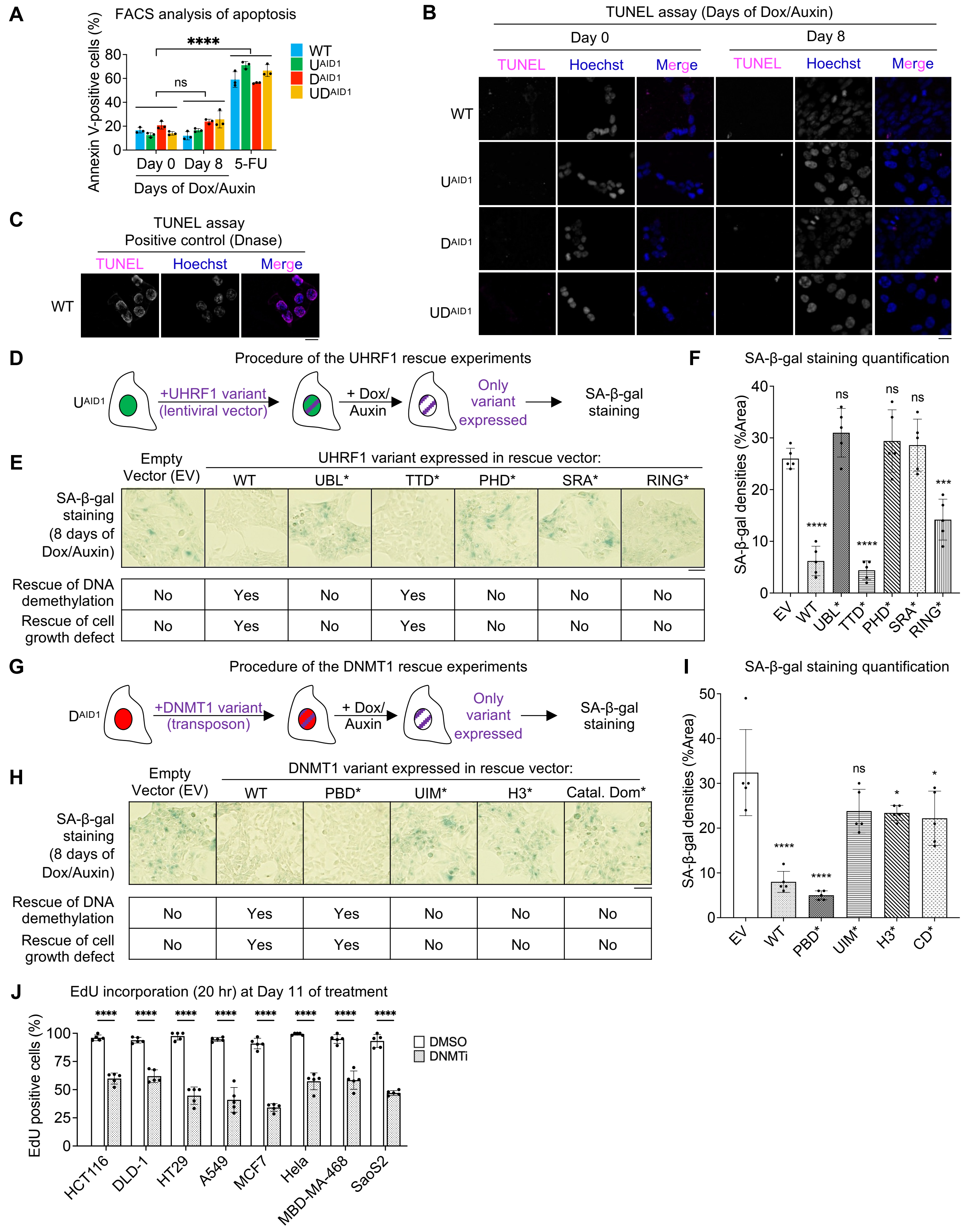

Supplementary Fig. 1: Lack of apoptosis in cells depleted of UHRF1 or DNMT1; rescue experiments support the role of DNA methylation maintenance in preventing senescence.

(A) Percentages of apoptotic cells scored by Annexin V positivity. N = 3 biological replicates. (B) Representative TUNEL assay images. Scale bar: 20 µm. (C) Positive control of TUNEL assay. Scale bar: 20 µm. (D) Schematic of UHRF1 rescue experiment. (E) Representative images of SA-β-gal staining with UHRF1-depleted HCT116 cells complemented by the indicated mutants. Scale bar: 50 µm. (F) Quantification of SA-β-gal staining. N = 5 fields of view. (G) Schematic of DNMT1 rescue experiment. (H) Representative images of SA-β-gal staining with DNMT1-depleted HCT116 cells complemented by the indicated mutants. Scale bar: 50 µm. (I) Quantification of SA-β-gal staining. N = 5 fields of view. (J) Quantification of EdU incorporation during a 20-hour pulse (related to Figure 1M). All data are presented as mean ± SD. Data of (A) are analyzed by two-way ANOVA test with Dunnett's multiple comparisons test. Data of (F) and (I) are analyzed by one-way ANOVA test with Dunnett's multiple comparisons test. Data of (J) are analyzed by two-way ANOVA test with Sidak's multiple comparisons test. In all figures we use the following convention: * $p < 0.05$, *** $p < 0.001$, **** $p < 0.0001$, ns: non-significant. Source data are provided as a Source Data file.

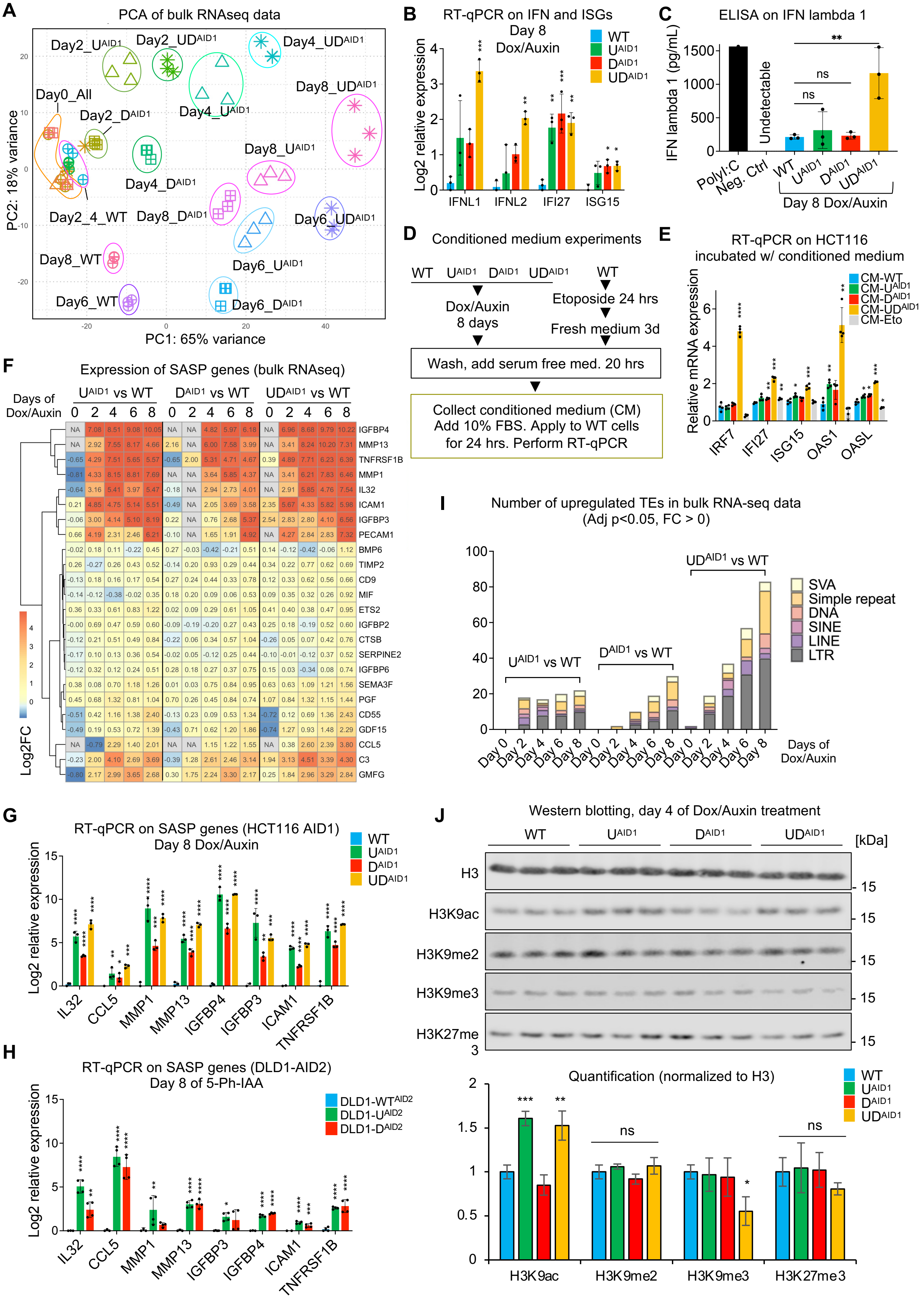

A
PCA of bulk RNAseq data
Day2_UAID1
Day2_UDAID1
Day4_UDAID1
Day8_UDAID1
Day0_All
Day2_DAID1
Day4_UAID1
Day4_DAID1
Day8_UAID1
Day2_4_WT
Day8_DAID1
Day6_UDAID1
Day8_WT
Day6_UAID1
Day6_WT
Day6_DAID1
PC2: 18% variance
PC1: 65% variance
B
RT-qPCR on IFN and ISGs
Day 8
Dox/Auxin
WT
UAID1
DAID1
UDAID1
Log2 relative expression
IFNL1
IFNL2
IFI27
ISG15
C
ELISA on IFN lambda 1
IFN lambda 1 (pg/mL)
Undetectable
ns
ns
**
PolyI:C
Neg. Ctrl
WT
UAID1
DAID1
UDAID1
Day 8 Dox/Auxin
D
Conditioned medium experiments
WT UAID1 DAID1 UDAID1
WT
Dox/Auxin 8 days
Etoposide 24 hrs
Fresh medium 3d
Wash, add serum free med. 20 hrs
Collect conditioned medium (CM) Add 10% FBS. Apply to WT cells for 24 hrs. Perform RT-qPCR
E
RT-qPCR on HCT116 incubated w/ conditioned medium
Relative mRNA expression
CM-WT
CM-UAID1
CM-DAID1
CM-UDAID1
CM-Eto
IRF7
IFI27
ISG15
OAS1
OASL
F
Expression of SASP genes (bulk RNAseq)
Days of Dox/Auxin
UAID1 vs WT
DAID1 vs WT
UDAID1 vs WT
0 2 4 6 8 0 2 4 6 8 0 2 4 6 8
NA 7.08 8.51 9.08 10.05 NA NA 4.82 5.97 6.18 NA 6.96 8.68 9.79 10.22 IGFBP4
NA 2.92 7.55 8.17 4.66 2.16 NA 6.00 7.58 3.99 NA 3.41 8.24 10.20 7.31 MMP13
-0.65 4.29 7.51 5.65 5.57 -0.65 2.00 5.31 4.71 4.67 0.39 4.89 7.71 6.23 6.39 TNFRSF1B
-0.81 4.33 8.15 8.81 7.69 NA NA 3.64 5.85 4.37 NA 3.41 6.21 7.83 6.46 MMP1
-0.64 3.16 5.41 3.97 5.47 -0.18 NA 2.94 2.73 4.01 NA 2.91 5.85 4.76 7.54 IL32
0.21 4.85 4.75 5.14 5.51 -0.49 NA 2.05 3.69 3.58 2.35 5.67 4.33 5.82 5.98 ICAM1
-0.06 3.00 4.14 5.10 8.19 0.22 NA 0.76 2.68 5.37 2.54 2.83 2.80 4.10 6.56 IGFBP3
0.66 4.19 2.31 2.46 6.21 -0.10 NA 1.65 1.91 4.92 NA 4.27 2.84 2.83 7.32 PECAM1
-0.02 0.18 0.11 -0.22 0.45 0.27 -0.03 -0.42 -0.21 0.51 0.14 -0.12 -0.42 -0.06 1.12 BMP6
0.26 -0.27 0.26 0.43 0.52 -0.14 0.20 0.93 0.29 0.44 0.34 0.22 0.77 0.62 0.69 TIMP2
-0.13 0.18 0.16 0.17 0.54 0.27 0.24 0.44 0.33 0.78 0.12 0.33 0.62 0.56 0.66 CD9
-0.14 -0.12 -0.38 -0.02 0.35 0.18 0.20 0.09 0.06 0.53 0.09 0.35 0.32 0.26 0.92 MIF
0.36 0.33 0.61 0.83 1.22 0.02 0.09 0.29 0.61 1.05 0.41 0.40 0.38 0.47 0.95 ETS2
-0.00 0.69 0.47 0.59 0.60 -0.09 -0.19 -0.20 0.27 0.43 0.25 0.18 -0.19 0.52 0.60 IGFBP2
-0.12 0.21 0.51 0.49 0.84 -0.22 0.06 0.34 0.57 1.04 -0.26 0.05 0.07 0.42 0.76 CTSB
-0.17 0.10 0.13 0.36 0.54 -0.02 0.05 -0.12 0.25 0.44 -0.10 -0.01 -0.12 0.37 0.52 SERPINE2
-0.12 0.32 0.12 0.24 0.51 0.28 0.18 0.27 0.37 0.75 0.15 0.03 -0.34 0.08 0.74 IGFBP6
0.38 0.08 0.70 1.18 1.26 0.01 0.03 0.75 0.84 0.95 0.63 0.63 1.09 1.21 1.56 SEMA3F
0.45 0.68 1.32 0.81 1.04 0.70 0.26 0.45 0.84 0.74 1.07 0.84 1.32 1.15 1.44 PGF
-0.51 0.42 1.16 1.38 2.40 -0.13 0.23 0.09 0.53 1.34 -0.72 0.12 0.69 1.36 2.43 CD55
-0.49 0.19 0.53 0.72 1.39 -0.43 0.71 0.62 1.20 1.86 -0.74 1.27 0.93 1.48 2.29 GDF15
NA -0.79 2.29 1.40 2.01 NA NA 1.15 1.22 1.55 NA 0.38 2.60 2.39 3.80 CCL5
-0.23 2.00 4.10 2.69 3.69 -0.39 1.28 2.61 2.46 3.14 1.94 3.13 4.51 3.39 4.30 C3
-0.80 2.17 2.99 3.65 2.68 0.30 1.75 2.24 3.30 2.17 0.25 1.84 2.96 3.29 2.84 GMFG
Log2FC
G
RT-qPCR on SASP genes (HCT116 AID1)
Day 8 Dox/Auxin
WT
UAID1
DAID1
UDAID1
Log2 relative expression
IL32
CCL5
MMP1
MMP13
IGFBP4
IGFBP3
ICAM1
TNFRSF1B
H
RT-qPCR on SASP genes (DLD1-AID2)
Day 8 of 5-Ph-IAA
DLD1-WTAID2
DLD1-UAID2
DLD1-DAID2
Log2 relative expression
IL32
CCL5
MMP1
MMP13
IGFBP3
IGFBP4
ICAM1
TNFRSF1B
I
Number of upregulated TEs in bulk RNA-seq data
(Adj p<0.05, FC > 0)
UAID1 vs WT
DAID1 vs WT
UDAID1 vs WT
SVA
Simple repeat
DNA
SINE
LINE
LTR
Day 0
Day 2
Day 4
Day 6
Day 8
Days of Dox/Auxin
J
Western blotting, day 4 of Dox/Auxin treatment
WT
UAID1
DAID1
UDAID1
[kDa]
H3
H3K9ac
H3K9me2
H3K9me3
H3K27me3
15
Quantification (normalized to H3)
WT
UAID1
DAID1
UDAID1
ns
ns
H3K9ac
H3K9me2
H3K9me3
H3K27me3

Supplementary Fig. 2: Senescence-associated transcriptional programs, cytokine secretion, and chromatin modifications after UHRF1 and/or DNMT1 depletion.

(A) Principal component analysis (PCA) of bulk RNA-seq data. (B) RT-qPCR of selected interferon (IFN) and interferon-stimulated genes (ISGs) at day 8 of Dox/Auxin treatment. Data are shown as log2 relative expression. N = 3 biological replicates. (C) Enzyme-linked immunosorbent assay (ELISA) on IFN lambda 1 in the serum-free medium of HCT116 lines upon Dox/Auxin treatment at Day 8 or HCT116 WT transfected with PolyI:C (1 μg/mL) as positive control or WT without Dox/Auxin treatment as negative control (Neg. Ctrl). N = 3 biological replicates in WT, U^{AID1}, D^{AID1} and UD^{AID1}. (D) Scheme of experiments with conditioned medium (CM). (E) RT-qPCR for selected ISGs in WT HCT116 cells incubated with CM from each condition, as well as CM from etoposide-treated cells (CM-Eto). N = 3 technical replicates. (F) Heatmap of senescence-associated secretory phenotype (SASP) gene expression (log2 fold change) from bulk RNA-seq comparing U^{AID1}, D^{AID1}, and UD^{AID1} to WT cells over time. Rows are grouped by hierarchical clustering. (G) RT-qPCR on selected SASP genes in HCT116 AID1 cells after 8 days of Dox/Auxin treatment. N = 3 biological replicates. (H) RT-qPCR on selected SASP genes in DLD1 AID2 degron cells at Day 8 of 5-Ph-IAA treatment. N = 2 biological replicates and 2 technical replicates. (I) Number of upregulated transposable elements (TEs) from bulk RNA-seq data across time points and conditions. TEs are categorized (SVA, simple repeat, DNA, SINE, LINE, LTR). (J) Western blot analysis of histone modifications at day 4 of Dox/Auxin treatment. Total H3 is shown as a loading control. Bar graph quantifies signal intensities normalized to H3. N = 3 biological replicates. Data of (B), (C), (E), (G), (H) and (J) are presented as mean ± SD and analyzed by one-way ANOVA test with Dunnett's multiple comparisons test. We use the following convention: * $p < 0.05$, ** $p < 0.01$, *** $p < 0.001$, **** $p < 0.0001$, ns: non-significant. Source data are provided as a Source Data file.

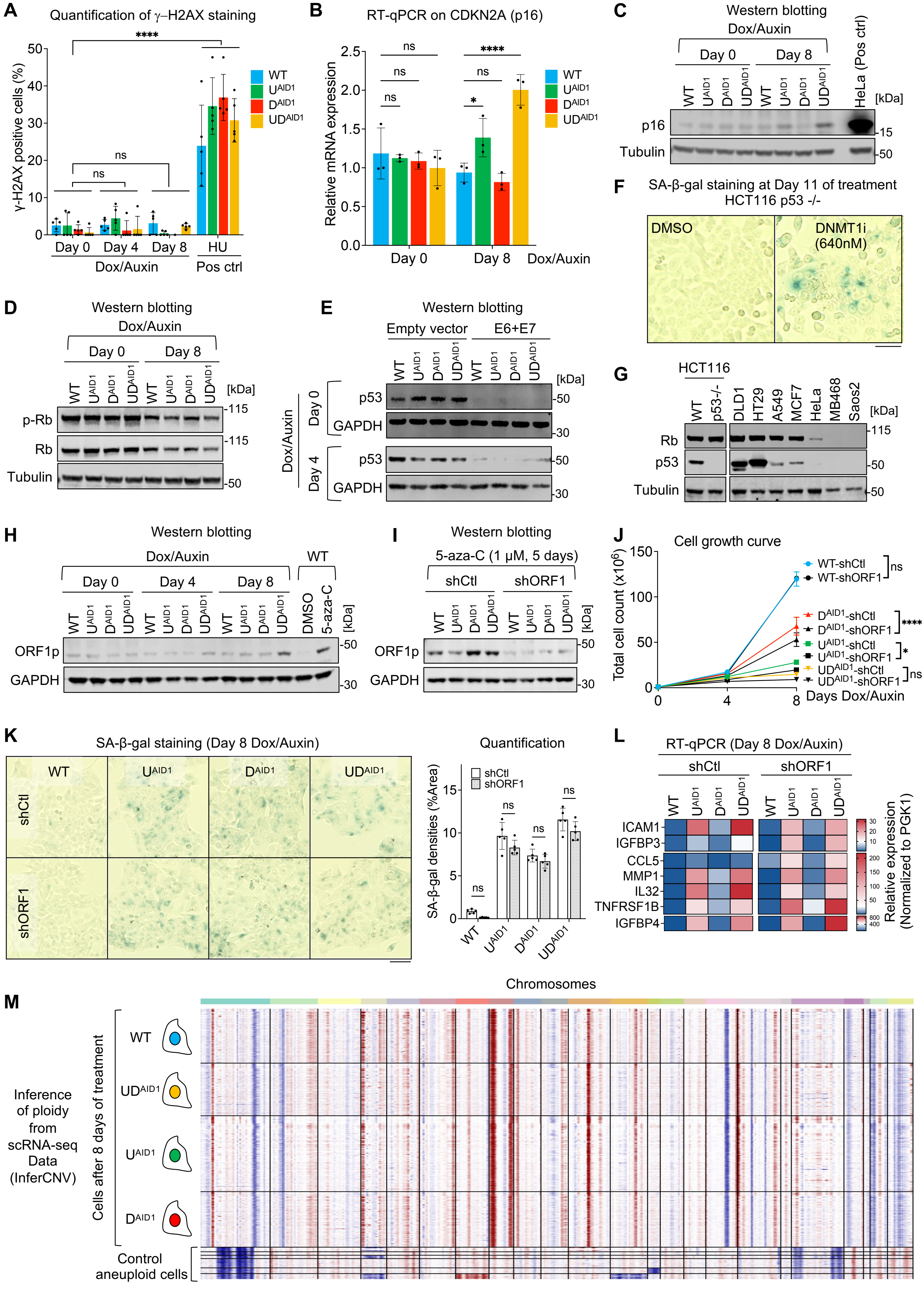

A
Quantification of γ–H2AX staining
γ-H2AX positive cells (%)
WT
UAID1
DAID1
UDAID1
ns
ns
****
Day 0
Day 4
Day 8
HU
Dox/Auxin
Pos ctrl
B
RT-qPCR on CDKN2A (p16)
Relative mRNA expression
ns
ns
ns
****
ns
*
Day 0
Day 8
Dox/Auxin
C
Western blotting
Dox/Auxin
Day 0
Day 8
HeLa (Pos ctrl)
[kDa]
p16
Tubulin
D
Western blotting
Dox/Auxin
p-Rb
Rb
Tubulin
E
Western blotting
Empty vector
E6+E7
p53
GAPDH
F
SA-β-gal staining at Day 11 of treatment
HCT116 p53 -/-
DMSO
DNMT1i (640nM)
G
HCT116
p53-/-
DLD1
HT29
A549
MCF7
HeLa
MB468
Saos2
H
Western blotting
Dox/Auxin
DMSO
5-aza-C
ORF1p
GAPDH
I
Western blotting
5-aza-C (1 μM, 5 days)
shCtl
shORF1
J
Cell growth curve
Total cell count (x10^6)
WT-shCtl
WT-shORF1
DAID1-shCtl
DAID1-shORF1
UAID1-shCtl
UAID1-shORF1
UDAID1-shCtl
UDAID1-shORF1
Days Dox/Auxin
K
SA-β-gal staining (Day 8 Dox/Auxin)
Quantification
SA-β-gal densities (%Area)
L
RT-qPCR (Day 8 Dox/Auxin)
ICAM1
IGFBP3
CCL5
MMP1
IL32
TNFRSF1B
IGFBP4
Relative expression (Normalized to PGK1)
M
Chromosomes
Inference of ploidy from scRNA-seq Data (InferCNV)
Cells after 8 days of treatment
Control aneuploid cells

Supplementary Fig. 3: DNA demethylation-induced senescence is independent of p53 and p16/Rb: further controls and quantifications; investigation of the role of L1 repeats and of aneuploidy.

(A) Quantification of the percentage of γ-H2AX foci positive cells in the indicated conditions. N = 5 fields of view. (B) RT-qPCR results of CDKN2A (p16) in indicated conditions. N = 3 biological replicates. (C) Immunoblots for p16 in indicated HCT116 lines upon Dox/Auxin treatment. HeLa cells used as positive control. (D) Immunoblots for total Rb and phosphorylated Rb (Ser807/811) in indicated conditions. (E) Immunoblots for p53 in indicated HCT116 lines upon Dox/Auxin treatment. (F) Representative images of SA-β-gal staining after treatment of HCT116 p53-/- cells with DMSO or DNMT1 inhibitor GSK-3685032 (640 nM). (G) Immunoblots for total Rb and p53 in the indicated cell lines. (H) Immunoblots for ORF1p (LINE-1 protein) in the indicated conditions. As a positive control, WT HCT116 cells were treated with 5-aza-cytidine (1 μM, 5 days). (I) Immunoblot validating the efficiency of the shORF1 vector. The indicated cells were infected with the shCtl or shORF1p vector, selected, then treated with 5-aza-cytidine (1 μM, 5 days). Extracts were then processed for immunoblotting. (J) Total cell numbers at the indicated days of Dox/Auxin treatment. N = 3 technical replicates. (K) SA-β-gal staining and quantification of indicated lines. N = 5 fields of view. (L) RT-qPCR on selected SASP genes in the indicated conditions. (M) Inference of large-scale chromosomal copy number variations (CNVs) using scRNA-seq data (inferCNV analysis). Heatmap shows inferred CNVs across genomic regions (x-axis) for individual cells (y-axis). Chromosomal gains and losses are represented in red and blue, respectively. HCT116 degron cell lines and control aneuploid cells shown in the lower portion of the heatmap exhibit widespread chromosomal alterations. Control aneuploid cells are derived from Massachusetts General Hospital (MGH) patient-derived malignant glioma samples (MGH36, MGH60, MGH53, MGH54, MGH93, and MGH97). Reference cells, derived from non-malignant populations such as microglia/macrophages and oligodendrocytes (Source Data), define the baseline gene expression and are assumed to be diploid. All scale bars are 50 μm. Data of (A) and (B) are presented as mean ± SD and analyzed by two-way ANOVA with Dunnett's multiple comparisons test. Data of (J) and (K) are presented as mean ± SD and analyzed by two-way ANOVA with Sidak's multiple comparisons test. Heatmap data of (L) are presented as mean from 3 technical replicates. In all figures we use the following convention: * $p < 0.05$, **** $p < 0.0001$, ns: non-significant. Source data are provided as a Source Data file.

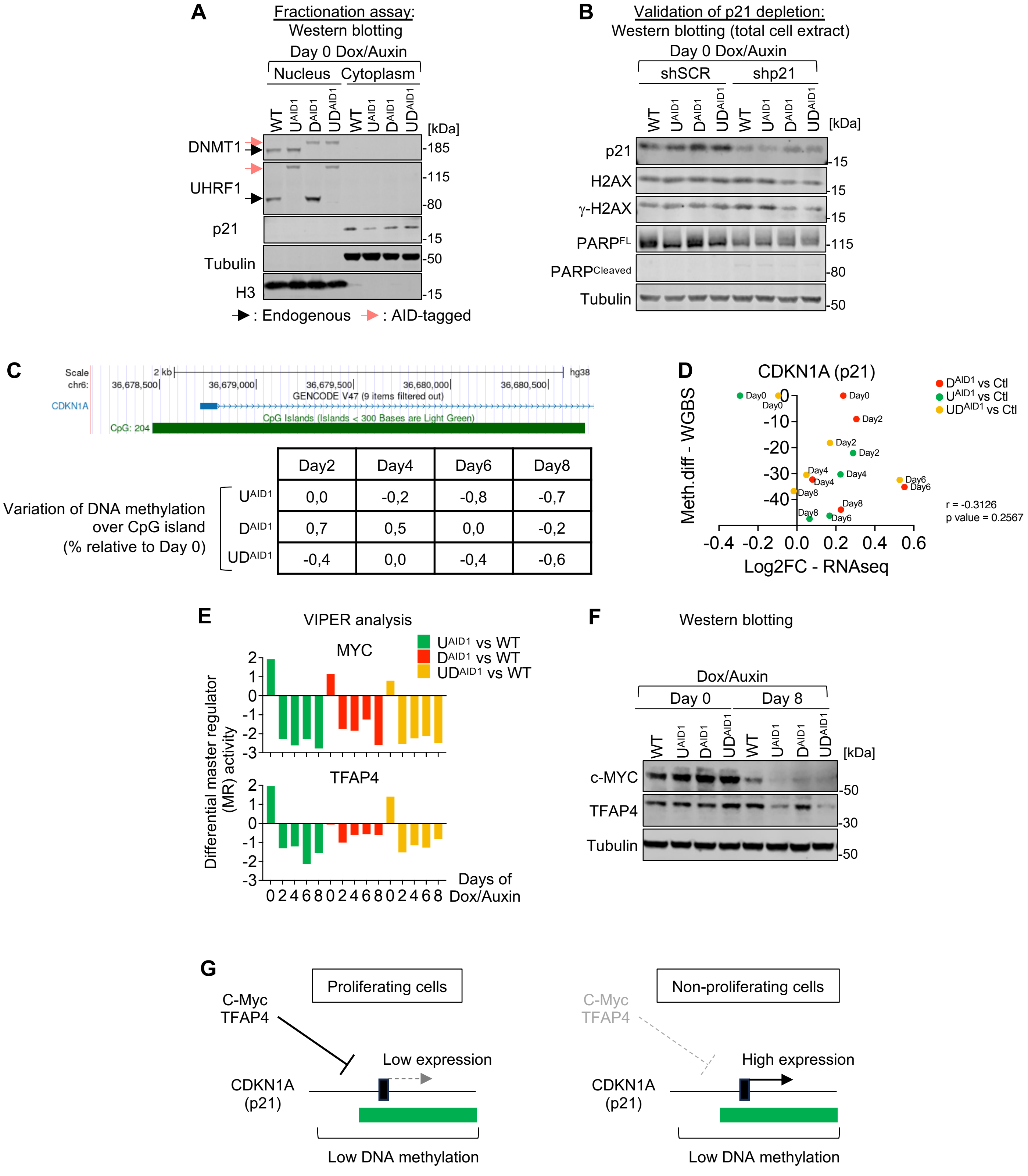

Supplementary Fig. 4: Further investigation of the MYC-TFAP4-p21 cascade.
(A) Immunoblots for DNMT1, UHRF1, p21 in the nuclear and cytoplasmic fractions of indicated lines at Day 0 of Dox/Auxin treatment. Black arrows indicate the endogenous proteins of interest and red arrows indicate the endogenous AID-tagged proteins of interest. (B) Immunoblots of p21, H2AX, γ-H2AX, full-length (FL) PARP and cleaved PARP in the indicated lines at Day 0 of Dox/Auxin treatment. (C) Representation of the CDKN1A/p21 CpG island and analysis of WGBS dataset. (D) Correlation analysis of DNA methylation on the CDKN1A (p21) promoter (-1200 bp to 300 bp) and expression of the CDKN1A (p21) mRNA. There is no correlation between expression and promoter DNA methylation. (E) Plots of the master regulator (MR) activity of c-Myc and TFAP4 using the VIPER (Virtual Inference of Protein-activity by Enriched Regulon analysis) algorithm at the indicated time points. (F) Immunoblots of c-Myc and TFAP4 in the indicated conditions. (G) Summary of the p21 regulation data. Source data are provided as a Source Data file.

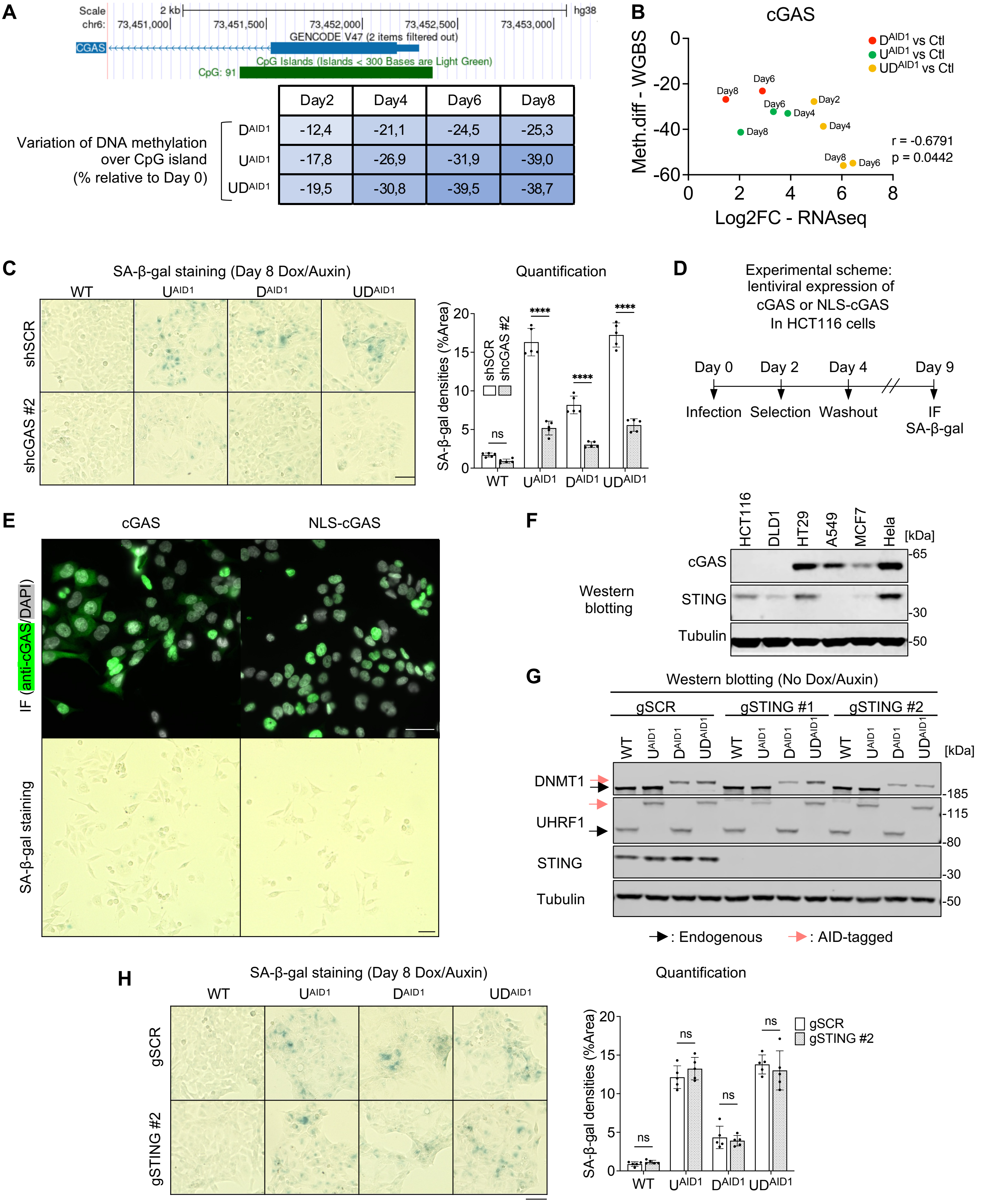

	Day2	Day4	Day6	Day8
D^{AID1}	-12,4	-21,1	-24,5	-25,3
U^{AID1}	-17,8	-26,9	-31,9	-39,0
UD^{AID1}	-19,5	-30,8	-39,5	-38,7

Supplementary Fig. 5: Additional experiments on cGAS and STING in senescence induced by loss of DNA methylation. (A) Representation of the cGAS CpG island and analysis of WGBS dataset. (B) Combined analysis of the WGBS and bulk RNA-seq data shows that induction of cGAS mRNA correlates with loss of promoter DNA methylation. The promoter is defined as the region covering 1200 bp upstream to 300 bp downstream of the TSS. (C) SA-β-gal staining (left panel) and quantification (right panel) of the indicated lines. N = 5 fields of view. (D) Experimental scheme for lentiviral expression of wild-type cGAS or nuclear-localized cGAS (NLS-cGAS) in HCT116 degron cells, followed by IF and SA-β-gal staining on Day 9. (E) Immunofluorescence (top) and SA-β-gal staining (bottom) on cells overexpressing cGAS (NB: the fields shown at top and bottom are different). (F) Western blotting of cGAS and STING in the indicated cancer cell lines. (G) Western blot analysis of DNMT1, UHRF1, and STING in the indicated lines untreated with Dox/Auxin. Black arrows indicate the endogenous protein of interest and red arrows indicate the endogenous AID-tagged protein of interest. (H) SA-β-gal staining (left panel) and quantification (right panel) of the indicated lines. N = 5 fields of view. All scale bars are 50 µm. Data of (C) and (H) are presented as mean ± SD and analyzed by two-way ANOVA with Sidak's multiple comparisons test. We use the following convention: **** $p < 0.0001$, ns: non-significant. Source data are provided as a Source Data file.

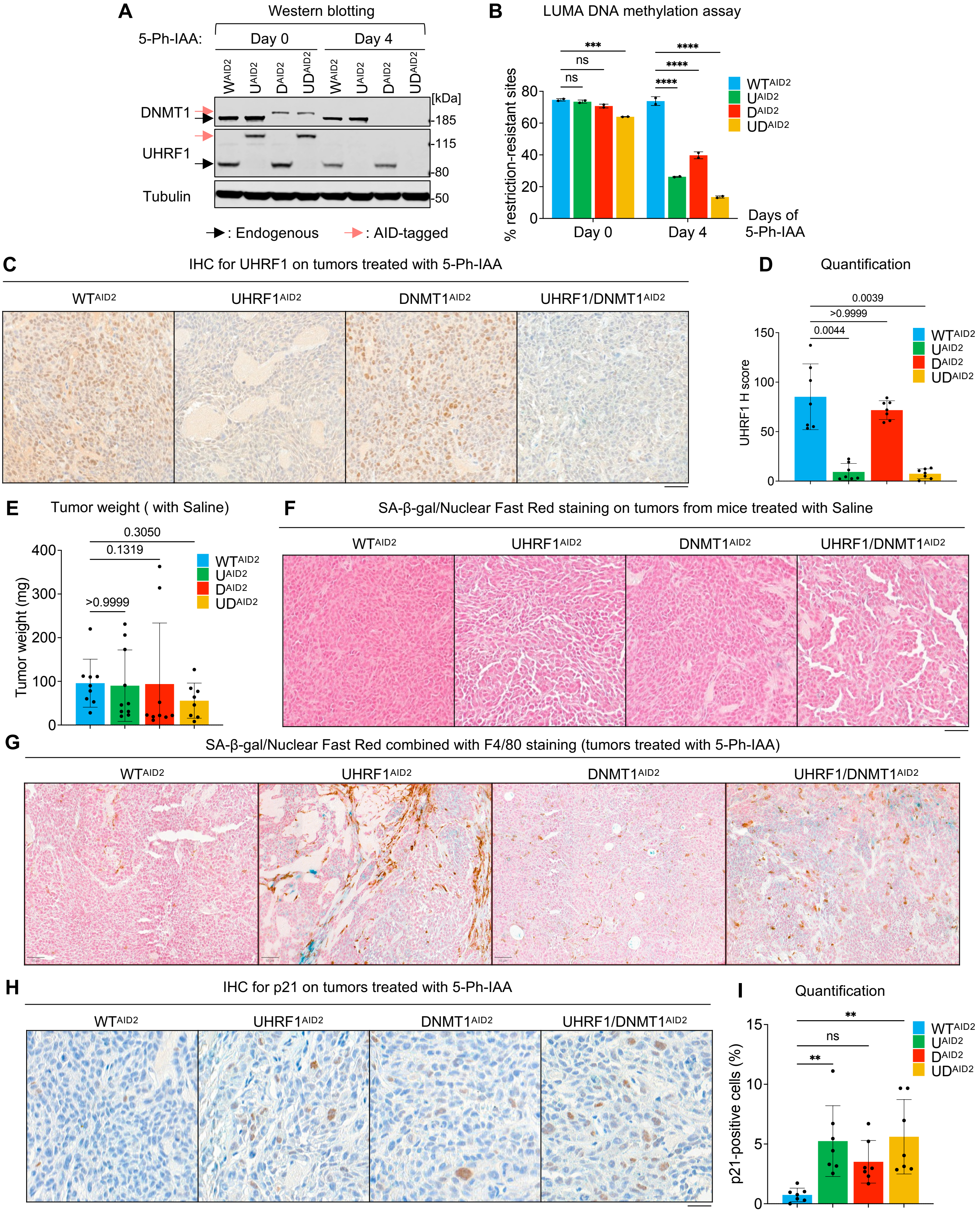

Supplementary Fig. 6: Validation and senescence assessment of HCT116 AID2 system *in vitro* and *in vivo*.
(A) Immunoblots of UHRF1 and DNMT1 in HCT116 AID2 lines with and without 5-Ph-IAA treatment. Black arrows indicate the endogenous proteins of interest and red arrows indicate the endogenous AID-tagged proteins of interest. (B) Quantification of the DNA methylation level in each HCT116 AID2 line by LUminometric Methylation Assay (LUMA). N = 2 biological replicates. (C) IHC staining of UHRF1 in the indicated tumors. (D) Quantification of UHRF1 IHC using H score. N = 7 fields of view at 400× original magnification. (E) Tumor weight in the control mice treated with Saline. N = 9 tumors for WT^{AID2}, 10 tumors for U^{ADI2}, 9 tumors for D^{AID2}, and 8 tumors for UD^{AID2}. (F) Representative images of SA-β-gal staining in the indicated tumors. Nuclear Fast Red was used for counterstaining. (G) Representative images of SA-β-gal staining combined with F4/80 IHC. Nuclear Fast Red was used for counterstaining. (H) IHC staining of p21 in the indicated tumors. (I) Quantification of p21-positive cells from (H). N = 7 fields of view at 400× original magnification. All scale bars are 50 µm. All data are presented as mean ± SD. Data of (B) are analyzed by two-way ANOVA and Sidak's multiple comparisons test. Data of (D), (E) and (I) are analyzed by Kruskal-Wallis test and Dunn's multiple comparison test. We use the following convention: ** $p < 0.01$, *** $p < 0.001$, **** $p < 0.0001$, ns: non-significant. Source data are provided as a Source Data file.
