# Supplementary material for: DNA methylation protects cancer cells against senescence
Source: Nat Commun. 2025 Jul 1;16:5901. doi: 10.1038/s41467-025-61157-7 (PMC12216915; doi:10.1038/s41467-025-61157-7)
Supplement: Supplementary file 3 — Description of Additional Supplementary Files [file 41467_2025_61157_MOESM3_ESM.pdf]

Description of Additional Supplementary Files

Supplementary Data 1. Differential gene expression results from RNA-seq analysis

Supplementary Data 2. Oligonucleotide sequences
