# Supplementary material for: DNA methylation protects cancer cells against senescence
Source: Nat Commun. 2025 Jul 1;16:5901. doi: 10.1038/s41467-025-61157-7 (PMC12216915; doi:10.1038/s41467-025-61157-7)
Supplement: Supplementary file 5 — Reporting Summary [file 41467_2025_61157_MOESM5_ESM.pdf]

nature portfolio

Corresponding author(s): Kosuke Yamaguchi
Pierre-Antoine Defossez

Last updated by author(s): May 20, 2025

Reporting Summary

Nature Portfolio wishes to improve the reproducibility of the work that we publish. This form provides structure for consistency and transparency in reporting. For further information on Nature Portfolio policies, see our Editorial Policies and the Editorial Policy Checklist.

Statistics

For all statistical analyses, confirm that the following items are present in the figure legend, table legend, main text, or Methods section.

n/a	Confirmed	
☐	☒	The exact sample size (*n*) for each experimental group/condition, given as a discrete number and unit of measurement
☐	☒	A statement on whether measurements were taken from distinct samples or whether the same sample was measured repeatedly
☐	☒	The statistical test(s) used AND whether they are one- or two-sided<br>*Only common tests should be described solely by name; describe more complex techniques in the Methods section.*
☒	☐	A description of all covariates tested
☐	☒	A description of any assumptions or corrections, such as tests of normality and adjustment for multiple comparisons
☐	☒	A full description of the statistical parameters including central tendency (e.g. means) or other basic estimates (e.g. regression coefficient) AND variation (e.g. standard deviation) or associated estimates of uncertainty (e.g. confidence intervals)
☐	☒	For null hypothesis testing, the test statistic (e.g. *F*, *t*, *r*) with confidence intervals, effect sizes, degrees of freedom and *P* value noted<br>*Give P values as exact values whenever suitable.*
☒	☐	For Bayesian analysis, information on the choice of priors and Markov chain Monte Carlo settings
☐	☒	For hierarchical and complex designs, identification of the appropriate level for tests and full reporting of outcomes
☐	☒	Estimates of effect sizes (e.g. Cohen's *d*, Pearson's *r*), indicating how they were calculated

Our web collection on statistics for biologists contains articles on many of the points above.

Software and code

Policy information about availability of computer code

Data collection

Bulk RNA sequencing data collection:
Illumina NovaSeq 6000 sequencer

WGBS data collection:
Illumina HiSeq X Ten sequencer

Single-cell RNA sequencing data collection:
Illumina NextSeq 2000 sequencer

Western blotting image collection:
LI-COR Image Studio Lite v5.2.5

Cell number collection:
BioRad automated cell counter

Microscopy image collection:
Leica DMI6000 (Leica Microsystems)
MetaMorph Leica v6.1

RT-qPCR data collection:
Applied Biosystems QuantStudio 6 Pro

Flow cytometry data collection:
Beckman Coulter Cytoflex SRT
CytExpert SRT v1.2.0

Histology data collection:
Bond Polymer Detection (Leica DS9800)

ELISA data collection:
AMR-100 Microplate Reader (Hangzhou Allsheng)

LUMA data collection:
Qiagen PyroMark Q24
PyroMark Q24 Software v2.0.8

Data analysis

Statistics analysis:
Prism 10.3.1
R v4.10

Microscopy analysis:
ImageJ v2.14.0

Sequencing data analysis:
R v4.1.0
inferCNV v1.22.0
Cell Ranger pipeline v 8.0
Scanpy v1.11.0
clusterProfiler package v4.6.2
BMap v1.0
bedtools v2.29.2
methylKit v1.20.0
FastQC v0.11.9
Trim Galore v3.0
STAR v2.6.1d
FeatureCounts v1.5.0-p3
DESeq2 v1.38.3
TEcount v2.2.3

GSEA analysis:
GSEA v4.3.2

FACS analysis:
FlowJo v10.4

For manuscripts utilizing custom algorithms or software that are central to the research but not yet described in published literature, software must be made available to editors and reviewers. We strongly encourage code deposition in a community repository (e.g. GitHub). See the Nature Portfolio guidelines for submitting code & software for further information.

Data

Policy information about availability of data

All manuscripts must include a data availability statement. This statement should provide the following information, where applicable:

- Accession codes, unique identifiers, or web links for publicly available datasets
- A description of any restrictions on data availability
- For clinical datasets or third party data, please ensure that the statement adheres to our policy

The bulk RNA-seq data used and generated in this study have been deposited in the Gene Expression Omnibus (GEO) database under accession code GSE249536 (RNA-seq, https://www.ncbi.nlm.nih.gov/geo/query/acc.cgi?acc=GSE249536) and GSE278103 (https://www.ncbi.nlm.nih.gov/geo/query/acc.cgi?acc=GSE278103), respectively. The WGBS data used and generated in this study are available in the GEO database under accession code GSE236026 (https://www.ncbi.nlm.nih.gov/geo/query/acc.cgi?acc=GSE236026) and GSE278681 (https://www.ncbi.nlm.nih.gov/geo/query/acc.cgi?acc=GSE278681), respectively. The processed scRNA-seq data are available at Zenodo (https://doi.org/10.5281/zenodo.15105182).

Research involving human participants, their data, or biological material

Policy information about studies with human participants or human data. See also policy information about sex, gender (identity/presentation), and sexual orientation and race, ethnicity and racism.

Reporting on sex and gender	Sex and gender information have not been collected in this paper. Sex was not considered in the study design
Reporting on race, ethnicity, or other socially relevant groupings	The reporting on race, ethnicity, or other socially relevant groupings data was not used in this paper.
Population characteristics	The population characteristics were not used in this paper.
Recruitment	This paper did not include the data from recruitment of patient or volunteer.
Ethics oversight	This paper did not include human participants data to require ethics oversight.

Note that full information on the approval of the study protocol must also be provided in the manuscript.

Field-specific reporting

Please select the one below that is the best fit for your research. If you are not sure, read the appropriate sections before making your selection.

☒ Life sciences ☐ Behavioural & social sciences ☐ Ecological, evolutionary & environmental sciences

For a reference copy of the document with all sections, see nature.com/documents/nr-reporting-summary-flat.pdf

Life sciences study design

All studies must disclose on these points even when the disclosure is negative.

Sample size	Experimental sample sizes were chosen under widely accepted standard for in vitro and in vivo validation of tumor xenografts to achieve statistical significance. Similarly, groups sizes for in vitro experiments were selected based on prior knowledge of variations. The sample size for AID cell lines is generally 3 clones per group. The sample size for xenografts is 3 or 4 per group depending on the experiments. We however, made sure to repeat experiments as defined below to enable experimental and biological variability to be ascertained.
Data exclusions	No data was excluded.
Replication	We confirmed that all attempts to replicate the experiments were successful. The replicated experiments and analyses are:<br>- Cell lines validation for AID tagging and protein degradation, 3 biological replicates<br>- Cell lines validation for knockdown or knockout efficiency, 2 or 3 biological replicates<br>- SA-beta-gal staining for all conditions, 2 or 3 technical replicates<br>- RT-qPCR for all conditions, 3 technical replicates<br>- Measurement of DNA methylation by LUMA in each AID cell lines, 2 biological replicates and 2 technical replicates<br>- Bulk RNAseq analysis for each cell lines and time points, 3 biological replicates<br>- WGBS analysis for each cell lines and time points, 3 biological replicates<br>- Cell proliferation analysis in each AID cell lines, 3 biological replicates or 3 technical replicates<br>- FACS analysis and Annexin V analysis in all conditions, 3 biological replicates or 3 technical replicates<br>All data were repeated independently to ensure reproducibility. Data that include 3 biological replicates, each from at least 2 independent experiments with similar results. Where representative images are shown, experiments were repeated at least three times unless otherwise noted.
Randomization	Randomization was done wherever possible for in vitro and in vivo experiments. HCT116 AID2 cell lines and STING KO lines were selected randomly to make single clone cell line. Knockdown experiments, infected cells were selected randomly with antibiotic marker from bulk population. The mice were randomly selected before tumor implantation.
Blinding	Blinding was used for histology data analysis. All other signal and images were obtained by automated method, so the authors did not do any manual measurement.

Reporting for specific materials, systems and methods

We require information from authors about some types of materials, experimental systems and methods used in many studies. Here, indicate whether each material, system or method listed is relevant to your study. If you are not sure if a list item applies to your research, read the appropriate section before selecting a response.

Materials & experimental systems

n/a	Involved in the study
[ ]	[x] Antibodies
[ ]	[x] Eukaryotic cell lines
[x]	[ ] Palaeontology and archaeology
[ ]	[x] Animals and other organisms
[x]	[ ] Clinical data
[x]	[ ] Dual use research of concern
[x]	[ ] Plants

Methods

n/a	Involved in the study
[x]	[ ] ChIP-seq
[ ]	[x] Flow cytometry
[x]	[ ] MRI-based neuroimaging

Antibodies

Antibodies used

For primary antibodies of Western blots:
anti-UHRF1, Abcam, ab57083, 1/1000
anti-DNMT1, Cell Signaling Technology, #5032, 1/1000
anti-Tubulin, Abcam, ab7291, 1/1000
anti-c-MYC, Cell Signaling Technology, #5605, 1/1000
anti-TFAP4, Proteintech, 12017-1-AP, 1/1000
anti-p53, Cell Signaling Technology, #2524, 1/1000
anti-p21, Cell Signaling Technology, #2946, 1/200
anti-p16-INK4A, Proteintech, 10883-1-AP, 1/1000
anti-cGAS, Cell Signaling Technology, #15102, 1/500
anti-H2AX, Bethl, A300-083, 1/1000
anti-phospho-Histone H2A.X (Ser139), Sigma-Aldrich, #05-636, 1/1000
anti-ChK1, Cell Signaling Technology, #2360, 1/1000
anti-phospho-Chk1 (Ser345), Cell Signaling Technology, #2348, 1/1000
anti-ChK2, Cell Signaling Technology, #2662, 1/1000
anti-phospho-Chk2 (Thr68), Cell Signaling Technology, #2661, 1/1000
anti-GADPH, Abcam, ab9485, 1/1000
anti-Rb, Cell Signaling Technology, #9309, 1/1000
anti-phospho-Rb (Ser807/811), Cell Signaling Technology, #9308, 1/1000
anti-PARP, Cell Signaling Technology, #9542, 1/1000
ant-Histone H3, Abcam, ab1791, 1/1000
anti-H3K9ac, Cell Signaling Technology, #9649, 1/1000
anti-H3K9me2, Active Motif, #39240, 1/1000
anti-H3K9me3, Diagenode, pAb-056-050, 1/1000
anti-H3K27me3, Active Motif, #61018, 1/1000
anti-STING, Cell Signaling Technology, #13647, 1/1000
anti-LINE-1 ORF1p, Sigma-Aldrich, MABC1152, 1/1000
For secondary antibodies of western blots:
IRDye 800CW Donkey anti-Rabbit IgG, #926-32213, LiCOR, 1/10000
IRDye 680CW Donkey anti-Mouse IgG, #926-68072, LiCOR, 1/10000
Peroxidase AffiniPure™ Donkey Anti-Mouse IgG (H+L), #711-035-150, Jackson Research, 1/10000
Peroxidase AffiniPure™ Donkey Anti-Rabbit IgG (H+L), #711-035-152, Jackson Research, 1/10000
For Immunohistochemistry:
anti-UHRF1, Abcam, ab57083, 1/75, pH 9
anti-p21, Cell Signaling Technology, #2946, 1/75, pH 6
anti- Ki67, Abcam, ab15580, 1/500, pH 6
anti- F4/80, Cell Signaling Technology, #70076, 1/200, pH 6
For Immunofluorescence:
anti-phospho-Histone H2A.X (Ser139), Sigma-Aldrich, #05-636, 1/200
anti-cGAS, Cell Signaling Technology, #79978, 1/200

Validation

anti-UHRF1, Abcam, ab57083, https://www.abcam.com/en-us/products/primary-antibodies/uhrf1-antibody-3a11-ab57083
anti-DNMT1, Cell Signaling Technology, #5032, https://www.cellsignal.com/products/primary-antibodies/dnmt1-d63a6-xp-rabbit-mab/5032?srsltid=AfmBOorVa8lKHqrx65lVcOvOo7dlxsjozn53I1hUnlYuF9SP8Q_Bu4OF
anti-Tubulin, Abcam, ab7291, https://www.abcam.com/en-us/products/primary-antibodies/alpha-tubulin-antibody-dm1a-loading-control-ab7291
anti-c-MYC, Cell Signaling Technology, #5605, https://www.cellsignal.jp/products/primary-antibodies/c-myc-d84c12-rabbit-mab/5605
anti-TFAP4, Proteintech, 12017-1-AP, https://www.ptglab.com/products/TFAP4-Antibody-12017-1-AP.htm
anti-p53, Cell Signaling Technology, #2524, https://www.cellsignal.com/products/primary-antibodies/p53-1c12-mouse-mab/2524?srsltid=AfmBOopWdByYsLUq4FhKGrUB2oLXgzsQGUkpZM-AKR9rXk-u3JYMWD6B
anti-p21, Cell Signaling Technology, #2946, https://www.cellsignal.com/products/primary-antibodies/p21-waf1-cip1-dcs60-mouse-mab/2946?srsltid=AfmBOor01UVpJVSAyVpgnMkg-BofvBE_JUYp_ykXfcGG1ElbW20QDukm

	anti-p16-INK4A, Proteintech, 10883-1-AP, https://www.ptglab.com/products/P16,P19-Antibody-10883-1-AP.htm<br>anti-cGAS, Cell Signaling Technology, #15102, https://www.cellsignal.com/products/primary-antibodies/cgas-d1d3g-rabbit-mab/15102?srsltid=AfmBOooA21kH_7zI4foMozfSuBs4bosyPRNOJwiMCdTeT5r_rmsgHdzT<br>anti-H2AX, Bethl, A300-083, https://www.fortislife.com/products/primary-antibodies/rabbit-anti-h2ax-antibody/BETHYL-A300-083<br>anti-phospho-Histone H2A.X (Ser139), Sigma-Aldrich, #05-636, https://www.sigmaaldrich.com/FR/en/product/mm/05636?srsltid=AfmBOorrB4l4_Qo1vclHKD3wCGB1u10t7ywJY9mNXB6wNqCEZ--MZfRZ<br>anti-ChK1, Cell Signaling Technology, #2360, https://www.cellsignal.com/products/primary-antibodies/chk1-2g1d5-mouse-mab/2360?srsltid=AfmBOopSBCSp8bedZj10-rMHM6NtzzOW-zguc5v7y6mcDBp3C19JX6BU<br>anti-phospho-Chk1 (Ser345), Cell Signaling Technology, #2348, https://www.cellsignal.com/products/primary-antibodies/phospho-chk1-ser345-133d3-rabbit-mab/2348<br>anti-ChK2, Cell Signaling Technology, #2662, https://www.cellsignal.com/products/primary-antibodies/chk2-antibody/2662<br>anti-phospho-Chk2 (Thr68), Cell Signaling Technology, #2661, https://www.cellsignal.com/products/primary-antibodies/phospho-chk2-thr68-antibody/2661<br>anti-GADPH, Abcam, ab9485, https://www.abcam.com/en-us/products/primary-antibodies/gapdh-antibody-loading-control-ab9485<br>anti-Rb, Cell Signaling Technology, #9309, https://www.cellsignal.com/products/primary-antibodies/rb-4h1-mouse-mab/9309<br>anti-phospho-Rb (Ser807/811), Cell Signaling Technology, #9308, https://www.cellsignal.com/products/primary-antibodies/phospho-rb-ser807-811-antibody/9308<br>anti-PARP, Cell Signaling Technology, #9542, https://www.cellsignal.com/products/primary-antibodies/parp-antibody/9542<br>ant-Histone H3, Abcam, ab1791, https://www.abcam.com/en-us/products/primary-antibodies/histone-h3-antibody-nuclear-marker-and-chip-grade-ab1791<br>anti-H3K9ac, Cell Signaling Technology, #9649, https://www.cellsignal.com/products/primary-antibodies/acetyl-histone-h3-lys9-c5b11-rabbit-mab/9649<br>anti-H3K9me2, Active Motif, #39240, chrome-extension://efaidnbmnnnibpcajpcglclefindmkaj/https://www.activemotif.com/documents/tds/39239.pdf<br>anti-H3K9me3, Diagenode, pAb-056-050, https://www.diagenode.com/en/p/h3k9me3-polyclonal-antibody-classic-50-ug<br>anti-H3K27me3, Active Motif, #61018, chrome-extension://efaidnbmnnnibpcajpcglclefindmkaj/https://www.activemotif.com/documents/tds/61017.pdf<br>anti-STING, Cell Signaling Technology, #13647, https://www.cellsignal.com/products/primary-antibodies/sting-d2p2f-rabbit-mab/13647<br>anti-LINE-1 ORF1p, Sigma-Aldrich, MABC1152, https://www.sigmaaldrich.com/FR/en/product/mm/mabc1152?srsltid=AfmBOopy7wfubWQXKqDjArUvRt3vYOZXEMf4A7oV9-JpVX-FAHFkjnw0<br>anti-cGAS, Cell Signaling Technology, #79978, https://www.cellsignal.com/products/primary-antibodies/cgas-e5v3w-rabbit-mab/79978<br>anti-p21, Cell Signaling Technology, #2946, https://www.cellsignal.com/products/primary-antibodies/p21-waf1-cip1-dcs60-mouse-mab/2946?srsltid=AfmBOor01UVpJVSAyVpgnMkg-BofvBE_JUYp_ykXfcGG1ElbW20QDukm<br>anti- Ki67, Abcam, ab15580, https://www.abcam.com/en-us/products/primary-antibodies/ki67-antibody-ab15580<br>anti- F4/80, Cell Signaling Technology, #70076, https://www.cellsignal.com/products/primary-antibodies/f4-80-d2s9r-xp-rabbit-mab/70076

Eukaryotic cell lines

Policy information about cell lines and Sex and Gender in Research

Cell line source(s)	HCT116 cell line (Tet-inducible OsTIR1) was obtained form RIKEN BRC Cell Bank. HCT116 and DLD1 cell line (constitutiely expressing OsTIR1- F74G) were generated from MTK lab. AID-tagged cell lines, exogenous UHRF1 expressing cell lines, exogenous DNMT1 expressing cell lines, STING knockout cell lines, p16/p21/cGAS knockdown, cGAS/NLS-cGAS OE and E6/E7 OE cell lines were generated in PAD lab during the course of this study. Human cancer cell lines including HT29 (female), A549 (male), MCF7 (female), HeLa (female), MDA-MB-468 (female), SaoS2 (female) were acquired from the ATCC. The HCT116 p53 null (p53 -/-) cell line was a gift from Bert Vogelstein.
Authentication	AID tagging UHRF1 and DNMT1 was confirmed by PCR, western blot, and Sanger sequencing. Knockout for STING was validated by PCR and western blot. Knockdown for p16/p21/cGAS was validated by RT-qPCR and/or western blot. Overexpression of E6-E7, cGAS and NLS-cGAS was validated by RT-qPCR, IF and/or western blot. Parental cell lines (HCT116 and DLD1) and other cell lines (HT29, A549, MCF7, HeLa, MDA-MB-468 and SaoS2) were authenticated by Eurofins genomics provided STR analysis service.
Mycoplasma contamination	All cell lines tested negative for mycoplasma.
Commonly misidentified lines (See ICLAC register)	No commonly misidentified cell lines were used in this study.

Animals and other research organisms

Policy information about studies involving animals; ARRIVE guidelines recommended for reporting animal research, and Sex and Gender in Research

Laboratory animals	Six-week-old female Rj:NMRI-Foxn1 nu/nu mice were acquired from Janvier Labs and maintained under standard conditions (standard diet and water ad libitum) at 23 °C with 12 h light and 12 h dark cycles, in a specific pathogen-free (SPF) animal facility
Wild animals	The study did not involve wild animals.

Reporting on sex	As sex differences were not the focus of this study, we used only female mice to avoid any aggressive behavior of males, allowing to obtain more stable group housing, less stress and reducing variability in line with the 3Rs principle.
Field-collected samples	The study did not involve samples collected from the field.
Ethics oversight	Animal work was conducted according to the ethical guidelines applicable in France, in a duly authorized animal facility (Plateforme du Petit Animal du CRCL (P-PAC), agreement number D693880202). Our project received the agreement number APAFIS #49702-202404181412449 v3 from the French Ministry of Education and Research.

Note that full information on the approval of the study protocol must also be provided in the manuscript.

Plants

Seed stocks	*Report on the source of all seed stocks or other plant material used. If applicable, state the seed stock centre and catalogue number. If plant specimens were collected from the field, describe the collection location, date and sampling procedures.*
Novel plant genotypes	*Describe the methods by which all novel plant genotypes were produced. This includes those generated by transgenic approaches, gene editing, chemical/radiation-based mutagenesis and hybridization. For transgenic lines, describe the transformation method, the number of independent lines analyzed and the generation upon which experiments were performed. For gene-edited lines, describe the editor used, the endogenous sequence targeted for editing, the targeting guide RNA sequence (if applicable) and how the editor was applied.*
Authentication	*Describe any authentication procedures for each seed stock used or novel genotype generated. Describe any experiments used to assess the effect of a mutation and, where applicable, how potential secondary effects (e.g. second site T-DNA insertions, mosiacism, off-target gene editing) were examined.*

Flow Cytometry

Plots

Confirm that:

- [x] The axis labels state the marker and fluorochrome used (e.g. CD4-FITC).
- [x] The axis scales are clearly visible. Include numbers along axes only for bottom left plot of group (a 'group' is an analysis of identical markers).
- [x] All plots are contour plots with outliers or pseudocolor plots.
- [x] A numerical value for number of cells or percentage (with statistics) is provided.

Methodology

Sample preparation	To analyze the cell cycle, HCT116 cells were seeded in a 6-well plate and treated with Dox/Auxin for up to 8 days. The cells collected at Day 0, Day 4 and Day 8 were treated with 10 µM 5-bromo-2'-deoxyuridine (BrdU) (Sigma-Aldrich) for 1 hour at 37 °C in a CO2 incubator. Cells were washed, trypsinized, and resuspended in 750 µL of PBS before adding 2250 µL of ice-cold ethanol for fixation (yielding a final volume of 3 mL with 75% ethanol). The fixed cells were incubated for at least 30 min at -20 °C prior to flow cytometry analysis. Samples were denaturated in 2 N HCl for 15 min at room temperature, followed by two washes in PBS + 1% BSA. Samples were then incubated in 200 µL of anti-BrdU-FITC antibody (BD Bioscences) in PBS + 1% BSA for 60 min. After a washing step, samples were then incubated in PBS containing propidium iodide (PI) (1:500, Invitrogen) with 150 µg/mL RNAse A (Qiagen) overnight at 4 °C in the dark.<br>To analysis the Annexin V assay, cells adhered to the dishes and those detached in the supernatant were collected for apoptosis assays. Cell pellets were then resuspended in Annexin Binding buffer (10 mM HEPES, 140 mM NaCl, 2.5 mM CaCl2 pH 4). The following steps were performed using the Annexin V Conjugates for Apoptosis Detection kit (Invitrogen). Cells were stained with 0.1% (v/v) Annexin V and 1× Hoechst 33342 (Life Technologies) solution. Stained cell suspensions were incubated in the dark for 15 min at room temperature. 400 µL of 1× binding buffer was added to each tube and samples were then immediately analyzed by flow cytometry using the Beckman Coulter Cytoflex SRT.
Instrument	The flow cytometry was performed on the Beckman Coulter Cytoflex SRT. Data were collected by the CytExpert SRT v1.2.0 software.
Software	Data were analyzed by the FlowJo v10.4
Cell population abundance	For cell cycle analysis, we described S phase cells as BrdU signal positive cells. The G1, S and G2/M phases cells were described as BrdU signal negative cells. We seperated G1 and G2/M cells by DNA content (G1 = 2N, G2/M = 4N).<br>For Annexin V assay, we described Annexin V-negative and Annexin V-positive populations.
Gating strategy	The population was gated on size using SSC-A/FSC-A. To obtain single cell information, we used FSC-H/FSC-A gating. All gated information was supplied for cell population analysis.

- [x] Tick this box to confirm that a figure exemplifying the gating strategy is provided in the Supplementary Information.
